# A Novel Sensitive Method to Measure Catechol-O-Methyltransferase Activity Unravels the Presence of This Activity in Extracellular Vesicles Released by Rat Hepatocytes

**DOI:** 10.3389/fphar.2016.00501

**Published:** 2016-12-23

**Authors:** Enriqueta Casal, Laura Palomo, Diana Cabrera, Juan M. Falcon-Perez

**Affiliations:** ^1^Metabolomics Platform, CIC bioGUNE, CIBERehd, Bizkaia Technology ParkBizkaia, Spain; ^2^Exosomes Laboratory, Metabolomics Unit, CIC bioGUNE, CIBERehd, Bizkaia Technology ParkBizkaia, Spain; ^3^Ikerbasque, Basque Foundation for ScienceBilbao, Spain

**Keywords:** COMT assay, SPE–UPLC–MS/MS method, catecholamine, norepinephrine, dopamine, diclofenac, exosomes, extracellular vesicles

## Abstract

There is a clear need for drug treatments to be selected according to the characteristics of an individual patient, in order to improve efficacy and reduce the number and severity of adverse drug reactions. One of the main enzymes to take into account in pharmacogenomics is catechol O-methyltransferase (COMT), which catalyzes the transfer of a methyl group from *S*-adenosylmethionine to catechols and catecholamines, like the neurotransmitters dopamine, epinephrine, and norepinephrine. Although, most of this enzyme is associated to intracellular vesicles, recently it has also been detected in extracellular vesicles secreted by hepatocytes and in serum circulating vesicles. COMT has implications in many neurological and psychiatric disorders like Parkinson's disease, chronic fatigue, pain response, schizophrenia, and bipolar disorders. Remarkably, genetic variations of COMT affect its activity and are associated to various human disorders from psychiatric diseases to estrogen-induced cancers. Consequently, the establishment of new methods to evaluate COMT activity is an important aspect to investigate the biology of this drug-metabolizing enzyme. Herein, we have developed a sensitive and selective method to determine COMT activity. We first optimized the activity in rat liver incubated with two different substrates; norepinephrine and dopamine. The enzymatically formed products (normetanephrine and 3-methoxytyramine, respectively) were extracted by solid-phase extraction using weak cation exchange cartridges, chromatographically separated, and detected and quantified using a mass spectrometer. The range of quantitation for both products was from 0.005 to 25 μg/mL. This methodology offers acceptable recovery for both enzymatic products (≥75%) and good accuracy and precision (≤15%). The lower limit of quantifications were 0.01 and 0.005 μM for 3-methoxytyramine and normetanephrine, respectively. Importantly, this sensitive assay was able to detect the presence of COMT activity in extracellular vesicles secreted by hepatocytes supporting a potential role of these vesicles in catecholamines and catecholestrogens metabolisms. In addition, the presence of COMT activity in extracellular vesicles opens new possibilities to develop tools to evaluate personalized drug response in a low invasive manner.

## Introduction

COMT (EC 2.1.1.6) is an enzyme that catalyzes the transfer of a methyl group from *S*-adenosyl-L-methionine to one of the hydroxyls of a catechol in the presence of Mg^2+^ (for review see Männistö and Kaakkola, 1999; Pihlavisto and Reenilä, 2002). COMT plays an important role in the metabolism of opioids, catechol drugs, catecholamine neurotransmitters, and catecholestrogens (Bell et al., 2015; Zhang et al., 2015). COMT can be found in most mammalian tissues (e.g., in brain; regulating the amounts of norepinephrine, dopamine, and epinephrine), with highest activities in liver, kidney, and intestinal tract, where COMT may modulate the dopaminergic tone.

The structural organization of the COMT gene has been reviewed in detail by Lundström et al. (1995). There is one single gene for COMT, which codes for both soluble COMT (S-COMT; 24 kDa) and membrane-bound COMT (MB-COMT; 28 kDa). S-COMT is located in the cytosol and MB-COMT is anchored to the rough endoplasmic reticulum (Ulmanen et al., 1997; Myöhänen et al., 2010). Although, the catalytic amino acid sequences of the two forms are identical, the kinetic properties and *in vitro* regioselectivity of the enzymes are different. MB-COMT has higher affinity for catechol substrates and for S-adenosyl-L-methionine coenzyme (Lotta et al., 1995). Interestingly, this enzyme has also been detected in extracellular vesicles (EVs) secreted by hepatocytes (Conde-Vancells et al., 2008) and in serum circulating vesicles (Rodríguez-Suárez et al., 2014) suggesting a possible role of these vesicles in the metabolism of catecholamine neurotransmitters and catecholestrogens metabolisms.

Various pharmacogenetics studies have revealed that some single nucleotide polymorphisms of COMT gene can be very important to drug personalized treatment. The most widely studied variant is a G to A nucleotide substitution resulting in an amino acid change from valine to methionine at codon 158 (Val158Met) decreases the thermal stability of the COMT protein, resulting in a two- to four-fold decrease in the enzymatic activity (Lachman et al., 1996; Zhang et al., 2009). Variations in the activity of COMT influence various human disorders from psychiatric diseases to estrogen-induced cancers (Cavalieri and Rogan, 2006; Cavalieri et al., 2006; Tunbridge et al., 2006). In addition, COMT is responsible for the metabolism of drugs, therefore, variability in its activity has been shown to modulate the efficacy of some drugs such as antidepressants and opioids (Ji et al., 2012; Kapur et al., 2014).

Thus, the interest in evaluating the intracellular and extracellular activity of COMT is increasing and reliable, sensitive, and rapid analytical COMT assays are highly demanded by clinician and pharmaceutical agencies in order to personalize drug administration and avoid adverse effects of drugs. In addition, for the measurement of COMT activity in various disease states or disorders, for the determination of structure–activity relationships of COMT or for the testing of efficacy of new COMT inhibitors candidates used to increase the efficacy of L-DOPA in the treatment of Parkinson's disease (Mueller, 2015).

A general COMT assay consists of the handling of the sample and incubation with the substrates followed by separation and detection of the reaction products. To date, a variety of COMT enzyme assays have been developed (Pihlavisto and Reenilä, 2002; Aoyama et al., 2005; Masuda et al., 2006; Hirano et al., 2007; Reenilä and Rauhala, 2010; Kimos et al., 2016). However, to our knowledge, none of them uses ultraperformance liquid chromatography–tandem mass spectrometry (UPLC–MS/MS). Herein, we have developed a method for the determination of COMT activity *in vitro* using norepineprine or dopamine as endogenous native substrates, which are metabolized to normetanephrine and 3-methoxytyramine, respectively. To develop a robust, sensitive and selective method we used rat liver extracts, and once optimized, the method was applied to evaluate COMT activity in a more challenging samples such as EVs secreted by hepatocytes under normal and diclofenac-treated conditions.

## Materials and methods

### Development of SPE-UPLC-MS method

#### Rat liver extract

The *in vitro* experiments described in this study were performed using six Wistar rat livers of 12-weeks obtained from Harlan Laboratories (Indianapolis, IN, USA). Livers were cut on dry ice and aliquoted into ~50 mg. Aliquots were homogenized with a Precellys homogenizer from Thermo Fisher Scientific Inc. (Waltham, MA, USA), using soft tissue homogenizing beads (CK14. Thermo Fisher Scientific Inc.). Each aliquot was homogenized in 2 × 20 s cycles at 6000 rpm in 500 μL of 50 mM sodium phosphate buffer (pH 7.4) containing 10% glycerol. Subsequently, the resulting homogenates were transferred to fresh tubes and centrifuged at 14,000 rpm for 30 min (4°C). A pooled sample, prepared by mixing equal aliquots of each liver extract, was used to optimize the UPLC–MS/MS method. The protein final concentration of the liver extracts was determined by Bradford assay (Bradford, 1976) (Bio-Rad Laboratories, Inc) using bovine serum albumin as standard (Thermo Fisher Scientific Inc). All the animal experimentation was conducted in accordance with the Spanish Guide for the Care and Use of Laboratory Animals^1^. Animal protocols were approved by the CIC bioGUNE Institute and the regional Basque Country ethical committee (ref. P-CBG-CBBA-3610).

#### COMT enzymatic assay

Mixtures consisted of liver extract (50 μg of protein) incubated with 2 mM MgCl_2_, 200 μM S-adenosyl-L-methionine (Abbott S.r.l. Campoverde, Italy) and the substrate 0.1 mM norepinephrine (Tsunoda et al., 2002; Bonifácio and Soares-da-Silva, 2003; Sigma-Aldrich Corp. St. Louis, MO, USA) or 2 mM dopamine (Levitt et al., 1982) (Dopamine hydrochloride. Sigma-Aldrich Corp.) in a final volume of 300 μL of 50 mM sodium phosphate buffer (pH 7.4). The enzymatic reaction was performed for 1 h at 37°C in a water bath. Standard stock solutions of norepineprine and dopamine were prepared in methanol/formic acid (99.9/0.1; v/v%) and the working solutions by appropriate dilution in phosphate buffer. *S*-adenosyl-L-methionine working solution was prepared directly in phosphate buffer. Enzymatic reaction was stopped by the addition of 1 mL of acetonitrile/water/formic acid (75/23.8/1.2; v/v/v%). Mixtures were shaken for 30 min at 4°C and centrifuged at 14,000 rpm for 30 min (4°C). The supernatant (1.1 mL) was reduced to dryness in a centrifugal vacuum concentrator (miVac concentrator. Genevac SP Scientific; Warminster PA, USA). Blank samples (liver extract without analyte) were prepared from heat inactivated (10 min at 95°C) extract liver (50 μg of protein), not-incubated and subjected to the treatment as previously described.

#### Sample preparation: solid phase extraction

Solid Phase Extraction (SPE) was used to process the samples prior to UPLC–MS/MS analysis. The SPE protocol included: pretreatment of the samples, conditioning, and equilibration of the cartridges, sample loading, washing, and elution of the compounds of interest (Peaston et al., 2010). SPE 3cc Oasis weak cation-exchange cartridges (60 mg sorbent, 30 μm particle size; Waters Corp., Milford, MA, USA) were conditioned with 2.5 mL of methanol followed by 2.5 mL of ammonium phosphate 10 mM (pH 6.5). Subsequently, samples reconstituted in 1 mL of ammonium phosphate 10 mM (pH 6.5) were run through the cartridges. The cartridges were then washed with 3 mL of water, 2 mL of methanol, and 2 mL of acetonitrile. The analytes of interest were eluted with 2 mL of 2% formic acid in acetonitrile/water (95/5; v/v%). The elution solvent was evaporated to dryness in a centrifugal vacuum concentrator and the residue was reconstituted in 300 μL of acetonitrile/water/formic acid (60/39.9/0.1; v/v/v%). Finally, a total volume of 2 μL was injected into the UPLC system.

The recoveries (or extraction efficiency of SPE method) of normetanephrine and 3-methoxytyramine, at 2.5 μM, were determined by comparisons of the peak areas of each of the analytes in pre-extracted spiked blank samples (*n* = 4) against post-extracted spiked blank samples (*n* = 4). Standard stock solutions of normetanephrine (DL-Normetanephrine hydrochloride. Sigma-Aldrich Corp.) and 3-methoxytyramine (3-Methoxytyramine hydrochloride. Sigma-Aldrich Corp.) were prepared in methanol/formic acid (99.9/0.1; v/v%) and the working solutions by appropriate dilution in ammonium phosphate 10 mM (pH 6.5; for pre-extracted spiked samples) and acetonitrile/water/formic acid (60/39.9/0.1; v/v/v%; for post-extracted spiked samples).

#### Identification and quantification of enzymatically formed normetanephrine and 3-methoxytyramine

For COMT assay, the substrates (norepinephrine and dopamine) and enzymatic products (normetanephrine and 3-methoxytyramine) were chromatographically separated using an Acquity UPLC system (Waters Corp.). The system was equipped with two binary solvent pumps, a cooled autosampler (4°C) with a 10 μL injection loop and a column oven (40°C). A 2.1 × 100 mm, 1.7 μm BEH amide column (Waters Corp.) was used for separation of the analytes. Samples were injected (V_load_ 2 μL) from deactivated total vials (Waters Corp.). For liquid chromatography experiments, acetonitrile (LC-MS Chromasolv grade; Riedel-de Haën, Seelz, Germany), formic acid (analytical grade; Sigma), ammonium formate (analytical grade; Sigma), and Milli-Q water obtained using a Millipore system (Bedford, MA, USA) were used. A gradient was developed to separate the analytes. Solvent A consisted of 99.5% water, 0.5% formic acid, and 20 mM ammonium formate while solvent B consisted of 29.5% water, 70% acetonitrile, 0.5% formic acid, and 1 mM ammonium formate. The gradient was as follows: from 5 to 50% A in 2.4 min, from 50 to 99.9% A in 0.2 min, constant at 99.9% A for 1.6 min, back to 5% A in 0.3 min, and constant at 5% A for 0.5 min. The flow rate was 0.25 mL/min.

A Synapt G2-HDMS Time-of- fight (ToF) mass spectrometer (Waters Corp.) was used for the detection of the analytes. The mass spectrometer was operated in electrospray positive mode. The following source parameters were used for the analysis: capillary voltage 2500 V, sample cone voltage 25 V, extraction cone voltage 5 V, desolvation temperature 450°C, source temperature 120°C, cone gas flow 5 L/h, and desolvation gas flow 600 L/h. Ion optics were tuned to a resolution of 22.000 (FWHM) for *m/z* 556. In order to guarantee mass accuracy a solution of Leucine-Enkefaline acquired from Sigma-Aldrich Corp. [2 μg/mL in acetonitrile/water (50/50; v/v%) and 0.1% formic acid] was infused into the source via a second electrospray probe at 10 μL/min and used for a lock mass. Every 30 s a lock mass scan was recorded for 0.5 s. Analyte spectra were automatically corrected for fluctuations in the lock mass. Initially, to develop the UPLC–MS method the mass spectrometer was operated in normal acquisition mode (50–1200 Da) with a 0.2 s scan time. Then, in order to reach an optimized Lower Limit of Quantification (LLOQ) for normetanephrine and 3-methoxytyramine, the mass spectrometer was operated in enhanced duty cycle mode. In this mode, the explosion of ion bunches from the transfer T-Wave™ collision cell and the pusher of the analyzer are synchronized so that the duty cycle is optimal for a given *m/z*. In this case, normetanephrine and 3-methoxytyramine were measured in scan functions optimized for *m/z* 166 and *m/z* 151, respectively. The time for these scan functions was 0.2 s.

With regard to data analysis, extracted ion traces were obtained for normetanephrine (*m/z* 166.086) and 3-methoxytyramine (*m/z* 151.075) with a 20 mDa window, subsequently smoothed (2 points, 2 iterations) and integrated with QuanLynx software (Waters Corp.). The COMT activity was expressed as nanomol of product formed in an hour per 50 μg of protein.

#### Evaluation of the method

##### Preparation of calibration curves and quality controls

Calibrators were prepared from stock solutions 10 mM normetanephrine and 10 mM 3-methoxytyramine in acetonitrile/water/formic acid (60/39.9/0.1; v/v/v%). These stocks were diluted to give a series of solutions (from 0.005 to 25 μM) that were used to reconstitute a blank pool at the step immediately prior to chromatography. Six replicates of quality controls samples at 1 and 10 μM were prepared following the S-adenosyl-L-methionine procedure to evaluate the accuracy and precision of the method.

##### Selectivity

The selectivity is the ability of the analytical method to discriminate the analyte of interest from interferences. The selectivity of the assay was assessed, using blank samples from six different livers. Based on guidance for industry documents (Guidance for Industry Bionalytical Method Validation, 2011; Guideline on bioanalytical method validation, 2011) an interfering peak should be <20% of the mean peak area for the LLOQ.

##### Matrix effect

The determination of ionization suppression (or enhancement) by matrix extracts is important as this phenomenon can severely affect the sensitivity and robustness of the analytical method. The effect of matrix was also evaluated for normetanephrine and 3-methoxytyramine in six different livers comparing peak areas of neat standards and standards spiked in blank samples prior to their injection into the UPLC system. Solutions of each compound (1 and 10 μM in acetonitrile/water/formic acid (60/39.9/0.1; v/v/v%) were directly injected (neat standards) or added to blank samples (spiked standards).

### Application of the method to evaluate COMT activity in intra- and extracellular vesicles

#### Sample preparation

##### Rat microsomes

For microsomes preparation, fresh or cultured rat hepatocytes were collected and centrifuged at 800 rpm 5 min at 4°C. The obtained pellet was resuspended in 0.5 ml of 100 mM phosphateTris-HCl buffer (pH 7.4). The suspension was transferred to a standard 2 mL tube containing 500 mg of soft tissue homogenizing beads (CK14; Thermo Fisher Scientific Inc.). The solution was lysed with a Precellys® 24 tissue homogenizer (Thermo Fisher Scientific Inc.) in 2 × 20 s cycles at 6000 rpm. The resulting homogenate was centrifuged at 15,000 × g during 15 min at 4°C. Supernatant was then ultracentrifuged at 100,000 × g during 60 min at 4°C. Resulting microsomal pellet was solubilized in 50 mM sodium phosphate buffer (pH 7.4) containing 10% glycerol. Protein concentration was determined by Bradford assay using bovine serum albumin as standard (Thermo Fisher Scientific Inc).

##### Extracellular vesicles

Production and isolation of EVs secreted by primary rat hepatocytes were performed as described in Conde-Vancells et al. (2008) and Rodríguez-Suárez et al. (2014). Briefly, rat hepatocyte suspension was centrifuged at 400 rpm 3 min and seeded in 24 collagen-coated dishes (150 mm) with collagen type I from rat tail (BD Biosciences. San Jose, CA) at 20 million cells per dish. Cells were cultured in complete Dulbecco's modified eagle medium (DMEM, Gibco, Life technologies, Heidelberg, Germany) supplemented with 10% (v/v) fetal bovine serum (Gibco), streptomycin (0.1 mg/L. Lonza, Walkersville, MD, USA), penicillin (100 U/mL. Lonza), and amphotericin-B (0.25 μg/mL, Lonza) for 4 h at 37°C and 5% CO_2_. Then, cells were washed twice with DPBS and incubated for 36 h in complete DMEM containing 25 mM HEPES (previously depleted from contaminating vesicles by overnight centrifugation at 100,000 × g, 4°C). The 24 collagen-coated dishes were divided in 2 groups (12 dishes each group) and incubated individually with no additives or with 400 μM diclofenac sodium (Sigma-Aldrich). After 36 h incubation, media was collected and EVs isolated. The 36 h-conditioned medium was centrifuged at 500 × g during 10 min to remove lifted cells and large cellular debris. The resultant supernatant was filtrated through a 0.22 μm pore size filter system (Corning. NY USA), and filtrated supernatant was ultracentrifuged 30 min at 10,000 × g 4°C to remove large vesicles. The resultant supernatant was ultracentrifuged at 100,000 × g 4°C during 75 min to sediment EVs (mostly exosomes and small microvesicles). The supernatant was completely removed, and the sediment washed with DPBS by repeating ultracentrifuged at 100,000 × g 4°C during 75 min. Final sediment containing the EVs was resuspended in a total volume of 0.25 ml of 50 mM sodium phosphate buffer pH 7.4 with 10% glycerol.

#### Assay COMT activity application in microsomes and hepatic extracellular vesicles

Mixtures containing protein extracts (50 μg) were incubated with 2 mM MgCl_2_, 200 μM S-adenosyl-L-methionine, and 0.1 mM norepinephrine in a final volume of 300 μL of 50 mM sodium phosphate buffer (pH 7.4). The enzymatic reactions were performed by 1 h at 37°C in a water bath. After incubation, the mixtures were processed according to the above condition for the SPE–UPLC–MS/MS analysis.

## Results

### Optimization method

#### Incubation conditions

To optimize the incubation conditions for measuring COMT enzymatic activity several parameters were evaluated including required supplements, pH, and temperature of the incubation mixture, incubation time, and stability of the substrates and products during the reaction period. For COMT activity, Mg^2+^ ions are essential, since they are coordinated to both of the catecholic hydroxyls, to a water molecule and to three amino acid residues in the catalytic site of COMT (Vidgren and Ovaska, 1997). Therefore, Mg^2+^ ions are routinely added to reaction mixtures at 2 mM MgCl_2_. COMT assays are performed under physiological conditions (pH 7.4 and temperature 37°C). For the independent measurement of MB-COMT and S-COMT activity different approaches have been employed (Tsunoda et al., 2002). These include separation of the two forms by differential centrifugation and use of two different substrate concentrations and pH. In the present work, to simplify the assay we have focused on the detection of MB-COMT activity and consequently the optimal conditions established for MB-COMT were used. Regarding the substrate, a wide variety of them are available (Männistö and Kaakkola, 1999; Tsunoda et al., 2002). In our case, two endogenous substrates (norepinephrine and dopamine) were used. The use of endogenous substrates provides relevant information on how COMT activity works *in vivo* compared to other methods using artificial substrates.

#### Sample preparation: SPE method development

Currently, protein precipitation is the most widely employed sample preparation technique in enzymatic assays, when to liquid chromatography–mass spectrometry is used. However, in the present work, only this technique was found be inappropriate. Deposit of the salts were found in the ion source and cone using only this kind of extraction, which brought about the decrease of sensitivity and everyday clearance as well as column damage. To avoid these problems, we decided to process the samples prior to UPLC–MS/MS analysis using SPE. SPE extraction has advantages over traditional extraction methods such as increased extraction efficiency, higher selectivity, or greater reproducibility. If the interferences were removed as part of the sample preparation, then the analytes of interest could be analyzed with a more robust method. Thus, after incubation, enzymatically formed products (normetanephrine and 3-methoxytyramine) were isolated using weak cation-exchange bound silica cartridges. In this system, the main retention mechanism of the compounds of interest is based mainly on the electrostatic attraction of the charged functional group of the compounds to the charged group bonded to the silica surface. The weak cation-exchange SPE material contains an aliphatic carboxylic acid group bonded to the silica surface. This carboxylic acid group is a weak anion and has a p*K*_a_ of about 4.8. It will be negatively charged in solutions of at least 2 pH units above this value, and will isolate cations if the pH is one at which they are both charged. To improve ion exchange interactions on weak cation-exchange, in the case of cationic compounds the pH should be at least 2 pH units below the p*K*_a_ (normetanephrine and 3-methoxytyramine have a p*K*_a_ of 9.1 and 9.6, respectively). At low pH, the sorbent will be un-ionized, which shuts off the ion-exchange mechanism. Therefore, an acidic solution (2% formic acid) was used to elute the analytes of interest (normetanephrine and 3-methoxytyramine). The recoveries (or extraction efficiency of SPE method) of normetanephrine and 3-methoxytyramine were 75 and 82%, respectively. These recoveries were in the acceptable range for routine LC-MS analysis^2^.

#### UPLC–MS/MS method performance

We used an amide column to separate norepinephrine and normetanephrine and dopamine and 3-methoxytyramine. This column provides enhanced peak symmetry and strong retention characteristics for these highly polar low molecular weight bases (Grumbach and Fountain, 2010). Considering the strong dependence of chromatographic behavior on the mobile phase buffer, different concentrations of ammonium formate were tested for their effect on the retention of the analytes (data not shown). In the absence of ammonium formate the peak shapes were broad and unregulated. The pH value of the buffer may also have influence on the chromatographic behavior of analytes. 0.5% formic acid was used; under these conditions, the analytes were positively charged and the substrates and the products were completely separated (Table 1).

**Table 1:** **COMT enzymatic assay: substrates and products**.

**Compound**	**Structure and formula**	**[M + H]^+^**	**Base peak (m/z)**	**t_*r*_ min**
Dopamine	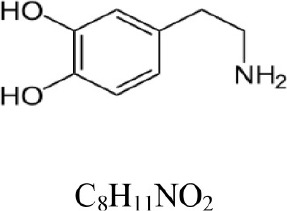	154.086	137.063 [M+H-NH_3_]^+^	1.3
3-Methoxytyramine	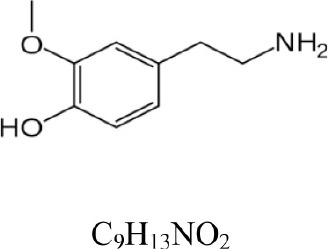	168.102	151.075 [M+H-NH_3_]^+^	1.1
Norepinephrine	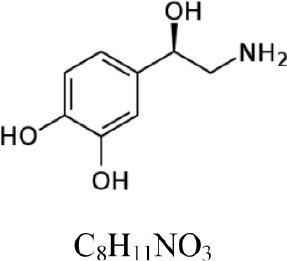	170.081	152.071 [M+H-H_2_O]^+^	1.6
Normetanephrine	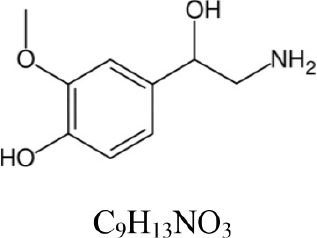	184.097	166.086 [M+H-H_2_O]^+^	1.3

**Figure 1:**
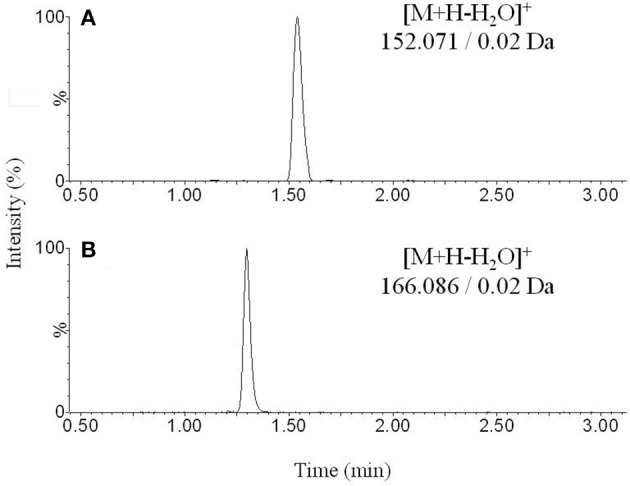
**Representative chromatograms obtained from blank sample spiked with (A)** norepinephrine and **(B)** normetanephrine.

**Figure 2:**
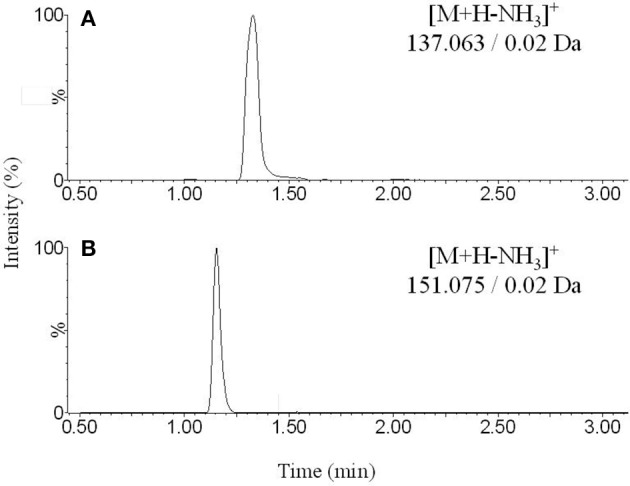
**Representative chromatograms obtained from extracted blank sample spiked with (A)** dopamine and **(B)** 3-methoxytyramine.

The Figures 1, 2 show the chromatograms of pairs norepinephrine/normetanephrine and dopamine/3-methoxytyramine, respectively. In the spectra of norepinephrine and normetanephrine the ions corresponding to [M+H-H_2_O]^+^ were represented as base peak, whereas in the case of dopamine and 3-methoxytyramine a loss of one molecule of ammonia was observed, forming the predominant ion [M+H-NH_3_]^+^ (Zhang et al., 2007; Peaston et al., 2010).

As part of method development, the evaluation of matrix effect on the quantitative analysis was estimated. Co-eluting matrix components, which are not observed in the chromatogram, can have a detrimental effect on the analysis. Matrix effect was assessed in different livers and no ion suppression (or ion enhancement) was observed.

The selectivity of the method was also evaluated and no peaks (interferences) were observed at the retention time of normetanephrine and 3-methoxytyramine. The good selectivity achieved could be due to several reasons. SPE procedure used was highly selective. In addition, the samples were analyzed by UPLC-MS/MS using a ToF mass spectrometer. UPLC was manufactured for the use of columns with sub-2 μm particles. These particles provide more resolution and more speed. Due to better resolution and more narrow peaks, analytes will co-elute less with interferences during ionization, so matrix effects could be lower, or even eliminated (Van De Steene and Lambert, 2008). The ToF mass spectrometer used is characterized by operating at a resolving power. Therefore, it gives accurate masses for both parent and fragment ions and enables the measurement of the elemental formula of a compound achieving compound identification. In addition, the tandem mass spectrometry provides more structural information, and enhances selectivity. Thanks to the robustness of the method, the use of internal standard was omitted and thus the method simplified.

With regard to calibration curves, power fitting was used to determine the relation between the signal (peak area) and sample concentration (see Table 2). The LLOQ, defined as the lowest concentration that can be measured with an acceptable level of accuracy (±20%) and a precision below 20%, is included in the Table 2.

**Table 2:** **Concentration range, equation and LLOQ of calibration curves**.

**Product**	**Range (μM)**	**Equation power regression**	**LLOQ (pmol)**
3-Methoxytyramine	0.01–25	[Signal] = 10,411x [concentration] ^0.923^ *R*^2^ > 0.99	0.02
Normetanephrine	0.005–25	[Signal] = 1177 × [concentration] ^0.833^ *R*^2^ > 0.99	0.01

The accuracy and precision were also assessed by determining quality control samples at two concentration levels. Accuracy was determined by comparing the mean observed concentration to the theoretical concentration and expressed as the ratio in percentage (% theoretical) and the precision by relative standard deviation (RSD%). In both cases, accuracy and precision were deemed to be acceptable (≤15%).

Test liver samples (*n* = 6) were injected in the same batch together with quality control and calibration standard samples. COMT activities in rat liver, incubated for 1 h with norepinephrine or dopamine as a substrate, produced 6.2 ± 0.1 ng of normetanephrine and 6.9 ± 0.1 ng of 3-methoxytyramine, respectively.

### Method application

COMT protein has been detected by proteomics and Western-blotting in extracellular vesicles (EVs) secreted by hepatocytes (Conde-Vancells et al., 2008) and in serum circulating vesicles (Rodríguez-Suárez et al., 2014) suggesting a possible role of these vesicles in the metabolism of catecholamine neurotransmitters and catecholestrogens metabolisms. However, in order to provide more evidence to this possibility it is very important to elucidate whether or not this enzyme is active in EVs. Here, we have applied our method to evaluate COMT activity associated to intracellular (microsomes) and extracellular (EVs) vesicles obtained from hepatocytes without treatment or treated with diclofenac (DCF) which is one of the most used over-the-counter anti-inflammatory worldwide, ranking in the top 10 causes of drug-induced liver injury^3^. Different samples were employed for this analysis: microsomes isolated from primary rat hepatocytes before seeding into dishes (Microsomes t0), microsomes isolated from primary hepatocytes after being cultivated for 36 h (Microsomes t36) with or without DCF, and extracellular vesicles secreted to the tissue culture media by primary hepatocytes incubated during 36 h (EVs t36) with or without DCF. All enzymatic determinations were performed in triplicates. Our method was able to detect COMT activity in all the samples including EVs (Table 3). The higher activity levels were detected in microsomes before putting the hepatocytes in culture. This microsomal activity was reduced 3 and 5 times by the 36 h of incubation of hepatocytes in the absence and presence of DCF, respectively (Table 3). This reduction indicates that during the 36-h incubation period COMT activity is reduced inside the cells; this effect is more severe in the case of the treatment with drug. Interestingly, we were able to detect COMT activity in EVs although the activity represented 1.1 and 2.3% of the microsomal activity in normal and DCF-treatment, respectively (Table 3).

**Table 3:** **COMT enzymatic activity in microsomes and extracellular vesicles obtained from primary rat hepatocytes**.

**Sample**	3-MT (nmol/mg/hour)^*^
*Microsomes t0*	78.80 ± 4.04
*Microsomes t36 Control*	24.70 ± 0.36
*Microsomes t36 Diclofenac*	13.80 ± 0.03
*EVs t36 Control*	0.27 ± 0.02
*EVs t36 Diclofenac*	0.32 ± 0.03

* *mean ± SD, n = 3*.

## Discussion

Traditionally, COMT activity has been measured by liquid chromatography coupled to different detectors: fluorescence, radiochemical, electrochemical, or UV detectors (Amorim-Barbosa et al., 2016). Although, these methods can be very sensitive, they could require derivatization and, moreover, possible interferences could be detected.

Mass spectrometry is a sensitive and powerful technology. To our knowledge, there is only a COMT assay which uses liquid chromatography coupled to mass spectrometry. In this assay, Mitamura et al. (2000) determined specifically the enzymatically formed guaiacol estrogens in rat brains by liquid chromatography coupled to ion trap mass spectrometer with atmospheric pressure chemical ionization and working in mode selected-ion monitoring. In our case, we have developed an alternative method to determine normetanephrine and 3-methoxytyramine, using another technology; in particular UPLC-electrospray ionization-time of flight analyzer working in tandem mass spectrometry mode. UPLC allows rapid analysis (5 min), for its part electrospray ionization is best suited to ionic compounds with high polarity and tandem mass spectrometry increases the specificity, sensitivity and throughput. In addition, we have used (prior to analytical chromatography) a double extraction with precipitation of protein and SPE. SPE has become the most powerful technique available for rapid, specific and selective sample preparation. SPE increases the sensitivity for concentrating of analytes, removes interferences to simplify chromatography and improves quantitation and protect the analytical column from contaminants. Although, we have performed the development of the method using weak cation exchange cartridges, this SPE is available in 96-well plates. This format is ideal for high throughput SPE allowing to process up to 96 samples in a couple of minutes.

The present assay would be potentially suitable for the detection of COMT activity not only in tissues, but also in other biological matrices such as cells, or plasma. In addition, although we have developed the method to detect specifically normetanephrine and 3-methoxytyramine, taking into account that weak cation-exchange SPE allows to concentrate strong bases and quaternary amines and that amide column was especially designed to retain polar compounds, other enzymatic substrates with chemical structures similar could be analyzed using this analytical method.

We have applied our method to evaluate COMT activity in intracellular and extracellular vesicles. We have previously shown by proteomics and Western-blotting analyses that COMT protein is present in EVs secreted by hepatocytes (Conde-Vancells et al., 2008) and also in serum circulating vesicles (Rodríguez-Suárez et al., 2014). Now, by using our method we were able to detect COMT activity in EVs secreted by hepatocytes. This activity associated to EVs seems to be increased when hepatocytes were treated with the drug diclofenac, which is one of the most frequently used over-the-counter anti-inflammatory worldwide, ranking in the top 10 causes of drug-induced liver injury. Although, the activity levels that we found associated to EVs were <3% of the intracellular activity, they could have a patho-physiological role that certainly requires further investigation.

There are increasing evidences that genetics variations of enzymes have a significant impact in endogenous and xenobiotics metabolisms what might mediate adverse effects of drugs. In order to minimize these adverse effects, there is a demand for drug treatments to be selected according to the characteristics of an individual patient. COMT enzyme is the most important enzyme in the metabolism of catecholamines, which are modulators of nervous and systemic responses. Our work, provide a sensitive, specific, and quick method that in combination with pharmacogenomics and pharmacometabolomics will be useful to define the different genetic and metabolic modifiers of this enzyme. Furthermore, the detection of COMT activity in extracellular vesicles able to circulate through blood stream open new possibilities to develop tools to evaluate personalized drug response in a low invasive manner.

As a conclusion, in this study we developed a highly specific and sensitive UPLC–MS/MS method and showed for the first time COMT activity associated with EVs.

## Author contributions

EC, LP, and DC performed the experiments. EC, LP, and JF performed the experimental design and write the manuscript.

### Conflict of interest statement

The authors declare that the research was conducted in the absence of any commercial or financial relationships that could be construed as a potential conflict of interest.

## Footnotes

^1^Royal Decree 1201/2005. Spanish Official State Gazette 252, 21st October 2005.

^2^Method validation and quality control procedures for pesticide residues analysis in food and feed (2012). Document N°SANCO/12495/2011.

^3^Livertox. *Clinical and Research Information on Drug Induced Liver Injury*. National library of medicine. Available online at: https://livertox.nlm.nih.gov//Diclofenac.htm
